# Optimization of a Focusable and Rotatable Shear-Wave Periodic Permanent Magnet Electromagnetic Acoustic Transducers for Plates Inspection

**DOI:** 10.3390/s17122722

**Published:** 2017-11-24

**Authors:** Xiaochun Song, Gongzhe Qiu

**Affiliations:** School of Mechanical Engineering, Hubei University of Technology, Wuhan 430068, China; 15623600353@163.com

**Keywords:** PPM EMATs, focusing, revolving, SH guided-wave, plate inspection

## Abstract

Due to the symmetry of conventional periodic-permanent-magnet electromagnetic acoustic transducers (PPM EMATs), two shear (SH) waves can be generated and propagated simultaneously in opposite directions, which makes the signal recognition and interpretation complicatedly. Thus, this work presents a new SH wave PPM EMAT design, rotating the parallel line sources to realize the wave beam focusing in a single-direction. The theoretical model of distributed line sources was deduced firstly, and the effects of some parameters, such as the inner coil width, adjacent line sources spacing and the angle between parallel line sources, on SH wave focusing and directivity were studied mainly with the help of 3D FEM. Employing the proposed PPM EMATs, some experiments are carried out to verify the reliability of FEM simulation. The results indicate that rotating the parallel line sources can strength the wave on the closing side of line sources, decreasing the inner coil width and the adjacent line sources spacing can improve the amplitude and directivity of signals excited by transducers. Compared with traditional PPM EMATs, both the capacity of unidirectional excitation and directivity of the proposed PPM EMATs are improved significantly.

## 1. Introduction

Compared to piezoelectric transducers, electromagnetic acoustic transducers (EMATs) can realize non-contact inspection based on electromagnetic coupling with the tested object, so they show some significant advantages when the sample to be inspected is hot, moving, or otherwise not suitable for a transducer to be placed on it directly [1]. Periodic-permanent-magnet electromagnetic acoustic transducers (PPM EMATs) can be used to generate and detect SH waves in non-ferromagnetic materials based on the Lorentz mechanism. Since SH waves have properties such as less reflection, beam steering, and attenuation, they are being used in an increasing number of nondestructive testing applications [2,3]. A typical PPM EMAT used to produce SH waves mainly consists of an array of magnets, which alternate in polarity with their nearest neighbors, and a racetrack coil that is excited with an alternating current [4,5,6]. It should be remembered that due to the symmetry of the transducer, two SH waves are produced, travelling in opposite directions to each other [7]. And the two waves can cause more end-reflection signals in echo signals of defects, it makes the signal recognition and interpretation complicatedly. Thus, it’s necessary to enhance the capacity of unidirectional excitation and the directivity of the transducers. Thompson [8] and Maxfield [9] investigated the method to excite unidirectional SH waves based on the constructive interference principle, namely a SH wave propagated in a certain angle can be excited by loading a narrowband signal with a particular frequency on the racetrack coil of PPM EMATs. Thus, changing the propagation angle of SH waves requires various excitation frequencies loaded. Namely, a broadband signal must be loaded on racetrack coils to create a wave-front that covers a large angular region [5,10], which may reduce the detection accuracy. In order to enhance the capacity of unidirectional excitation and the directivity of PPM EMATs, this work presents a new SH wave PPM EMATs design for inspecting plates, which can realize the wave beam focusing at single-direction without loading a broadband signal on racetrack coils. Changing angle between parallel line sources of the racetrack coil, the wave strength on the closing side can be improved and that on the opening side can be decreased. And adjusting the orientation of line sources by a mechanical device, a certain angular region can be scanned by SH waves. Because of the non-dispersion characteristic, SH_0_ wave generated by the proposed PPM EMATs, can be chosen to inspect the plate.

In this paper, Section 2 presents the theoretical model of a focusable and rotatable PPM EMAT based on the distributed line sources model. Section 3 describes the effects of such parameters as the inner coil width, adjacent line sources spacing and the angle between parallel line sources on SH wave focusing and directivity using 3D finite element model (FEM). Section 4 reports and analyzes the experimental measurement results of the proposed PPM EMATs.

## 2. Distributed Line Sources Models of PPM EMATs

### 2.1. The Far-field Response Induced by Single Line Source

**Figure 1:**
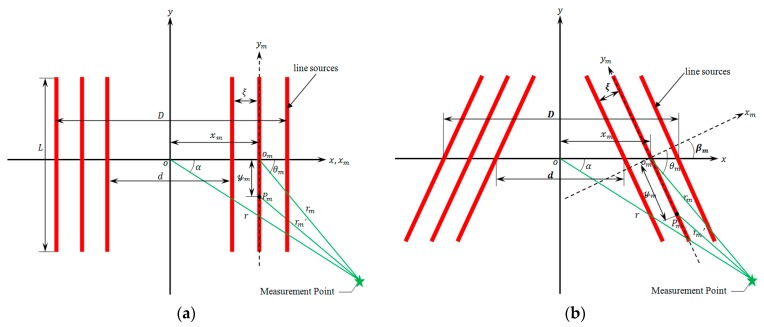
Distributed line sources models: (**a**) Conventional PPM EMATs; (**b**) Proposed PPM EMATs.

The distributed line sources model was originally proposed by Lee et al. [5]. As shown in Figure 1a, Ma et al. [11] set up the acoustic field model of PPM EMATs based on the model proposed by Lee et al. Introducing the local coordinate system $x_{m}o_{m}y_{m}$ for single line source m, the far-field response $R_{p}$ of harmonic point source located at $P_{m}(x_{m},y_{m})$ gives:

(1) $$R_{p}=\sqrt{\frac{2}{\pi kr_{m}{}^{\prime }}}e^{j\left(kr_{m}{}^{\prime }-\omega t+\frac{\pi }{4}\right)}$$

where $r_{m}^{\prime }$ is distance from $P_{m}$ to measurement point, ω is angular frequency, k is wave-number.

According to Huygens principle, the far-field response $R_{m}$ of single line source m can be integrated by the far-field response of point sources located on the line source along the length of the line source. Supposed that the source strength $R_{P}$ of harmonic point $P_{m}(x_{m},y_{m})$ located at the line source is *A_m_dy_m_*, where $A_{m}$ is a constant. The far-field response $R_{m}$ of s single line source m is:

(2) $$R_{m}\left(r_{m}{}^{\prime },\theta_{m},t\right)=\int_{-\frac{L}{2}}^{\frac{L}{2}}A_{m}R_{p}dy_{m}=A_{m}\int_{-\frac{L}{2}}^{\frac{L}{2}}\sqrt{\frac{2}{\pi kr_{m}{}^{\prime }}}e^{j\left(kr_{m}{}^{\prime }-\omega t+\frac{\pi }{4}\right)}dy_{m}$$

where $\theta_{m}$ is the included angle between the line stretching from the original point $o_{m}$ to the measurement point and $x_{m}$-axis in the local coordinate system $x_{m}o_{m}y_{m}$. As the measurement point is located at the far-field, it can be concluded that $r_{m}\gg L/2$, so the location of measurement point in far-field can be approximately presented as:

(3) $$r_{m}{}^{\prime }\approx r_{m}-y_{m}\sin\left(\theta_{m}\right)$$

Substituting Equation (3) into Equation (2), gives:

(4) $$\begin{array}{cc} R_{m}\left(r_{m},\theta_{m},t\right)\;\approx & A_{m}\int_{-\frac{L}{2}}^{\frac{L}{2}}\sqrt{\frac{2}{\pi k\left(r_{m}-y_{m}\sin\left(\theta_{m}\right)\right)}}e^{j\left(kr_{m}-ky_{m}\sin\left(\theta_{m}\right)-\omega t+\frac{\pi }{4}\right)}dy_{m}\\ & \approx A_{m}\sqrt{\frac{2}{\pi kr_{m}}}e^{j\left(kr_{m}-\omega t+\frac{\pi }{4}\right)}\int_{-\frac{L}{2}}^{\frac{L}{2}}e^{-j\left(ky_{m}\sin\left(\theta_{m}\right)\right)}dy_{m}\\ & \approx A_{m}L\sqrt{\frac{2}{\pi kr_{m}}}e^{j\left(kr_{m}-\omega t+\frac{\pi }{4}\right)}\left[\frac{sin\left(\frac{1}{2}kL\sin\left(\theta_{m}\right)\right)}{\frac{1}{2}kL\sin\left(\theta_{m}\right)}\right] \end{array}$$

Equation (4) is the far-field response of single line source m at measurement point $\left(r_{m},\theta_{m}\right)$. It should be noted that the coordinate of measurement point is represented in the local coordinate system $x_{m}o_{m}y_{m}$.

### 2.2. The Total Far-Field Response Induced by Distributed Line Sources

Compared to conventional PPM EMATs, the difference of the presented PPM EMAT is that the parallel line sources are rotated oppositely at an angle, shown in Figure 1b. By introducing the rotation angle of line sources $\beta_{m}$, the total far-field response of presented PPM EMATs is deduced on the basis of Equation (4). Taking the symmetry of the rotated line sources model into account, only the line sources on the right side are discussed. Suppose the number of line sources is M, summing $R_{m}$ for m=1 to M, the total response of measurement point (r,α) induced by distributed line sources gives:

(5) $$R\left(r,\alpha ,t\right)=\overset{M}{\underset{m=1}{\sum }}A_{m}L\sqrt{\frac{2}{\pi kr_{m}}}e^{j\left(\omega t-kr_{m}\right)}\left[\frac{\sin\left(\frac{1}{2}kL\sin\left(\theta_{m}\right)\right)}{\frac{1}{2}kL\sin\left(\theta_{m}\right)}\right]$$

with:

(6) $$\{\begin{array}{c} \begin{array}{c} r_{m}=\sqrt{{\left(r\sin\left(\alpha \right)\right)}^{2}+{\left(r\cos\left(\alpha \right)-x_{m}\right)}^{2}}\\ \theta_{m}=\arctan\left(\frac{r\sin\left(\alpha \right)}{r\cos\left(\alpha \right)-x_{m}}\right)+\beta_{m} \end{array}\\ x_{m}=\frac{d}{2}+\left(m-1\right)\times \xi \end{array}$$

where $\beta_{m}$ is the rotation angle of single line source m, $x_{m}$ is the horizontal distance between the original point o of global coordinate system xoy and the original point $o_{m}$ of local coordinate system $x_{m}o_{m}y_{m}$, d is the inner coil width, ξ is the spacing between adjacent line sources.

## 3. Effects of Some Line Sources Parameters on Performance of PPM EMATs

The main design parameters of PPM EMATs consist of the periodicity of magnet arrays, number of magnet arrays, inner coil width, spacing between adjacent line sources, sensors lift-off, and so on. Ma et al. [11] have investigated the effect of period and number of magnet arrays on the performance of PPM EMATs. Huang et al. [12] have demonstrated the effect of sensors lift-off on excitation signals. The biggest difference between the proposed PPM EMATs and the conventional ones is the change in the geometric configuration of the racetrack coils. Hence, in order to optimize the racetrack coil and obtain the optimal performance, the effects of some other parameters, such as the inner coil width, spacing between adjacent line sources and the rotation angle of line sources, on the amplitude and directivity of excitation signal are studied with the help of 3D FEM. A circle with a radius r = 100 mm, centered on the transducer, is made. The amplitudes of tangential displacement of the points on the circle at t = 3E − 5s are extracted to generate the radiation pattern. A line that passes the transducer center and extends toward the propagation direction of SH_0_ wave is constructed. The peak to peak amplitude of tangential displacements of two wave beams propagating in opposite directions along the line at t = 3E − 5s is extracted to generate the line graph. The directivity of the transducer is characterized by the amplitude ratio $R_{ms}$ between main lobe and side lobe in the radiation pattern. The capacity of unidirectional excitation of the transducer is characterized by the amplitude ratio $R_{co}$ between closing side and opening side in the line graph. The effective detecting distance of the transducer is characterized by the amplitude of the signal amplitude on the closing side in line graph.

### 3.1. FEM of Proposed PPM EMATs

**Figure 2:**
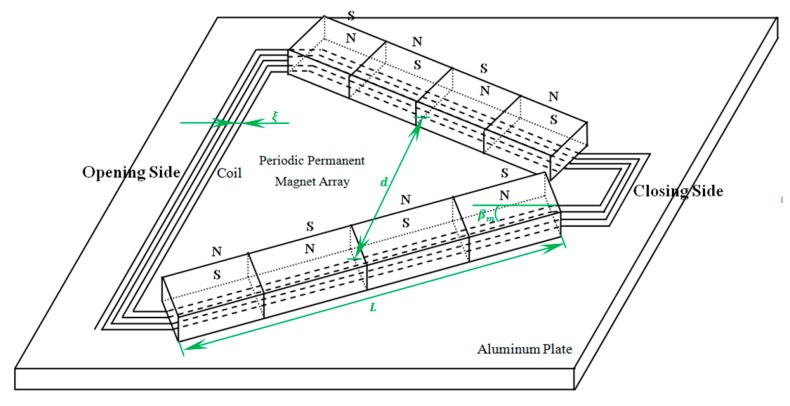
3D FEM of proposed PPM EMATs for inspecting plates.

With the use of COMSOL Multiphysics software, a 3D FEM shown in Figure 2, was designed for studying the influence of the coil parameters on the performance of PPM EMATs. The size and material parameters of the aluminum plate used in 3D FEM are shown in Table 1.

The 3D FEM process of the PPM EMAT proposed in this paper is similar to that of a torsional wave PPM EMAT array [2]. To decrease the computation cost, only one exciting unit is built in the 3D FEM model. The coil is modeled as round electric current whose diameter is 0.3 mm. There are five turns per coil, whose line sources is 20 mm long. The number array of magnets is two and the size of each permanent magnet is 8 mm × 6 mm × 10 mm, which alternate polarity with their nearest neighbors. The aluminum plate is defined as elastic region which is expressed by the pure elastic constitutive equation. In order to avoid the generation of reflected wave from defect, the elastic region is defined as infinite plate in simulation, and a layer of absorbing layers with increasing damping (ALID) has been used around the elastic region [13]. Although ALID has the same elastic properties as the original medium, there is also energy dissipation due to attenuation. Therefore, the dissipative properties due to attenuation are expressed by Rayleigh damping, namely:

(7) C=αM+βK

where C is the damping matrix, M is the mass matrix, K is the stiffness matrix, both α and β are the damping factor. In this paper, β=0, α is proportional to the cube of the distance r between the absorption domain and the origin (ie. $\alpha \propto r^{3}$) [14].

In order to obtain the static numerical solution, taking calculation load and precision into account, the number of elements are set as 15 per wave length and as 5 in the thickness direction, respectively. Since the variation of simulation results is not obvious while further subdividing the elements, it indicates that the model has been convergent with the present mesh settings. Based on the stability criterion such that $\Delta t<0.8\Delta x/C_{max}$(Δx is the element size and $C_{max}$ indicates the velocity of the wave that travels fastest through the material), an iteration step time with 1E-8s was used for the simulation. And the excitation signal is the sinusoidal modulated by five cycles of Hamming windows. In order to excite pure SH_0_ mode, the operation frequency f=200 KHz, phase speed of SH0 $c_{p}=3200\;m/s$, wave length of SH0 is λ=16 mm.

### 3.2. Effect of The Inner Coil Width on Performance of PPM EMATs

**Figure 3:**
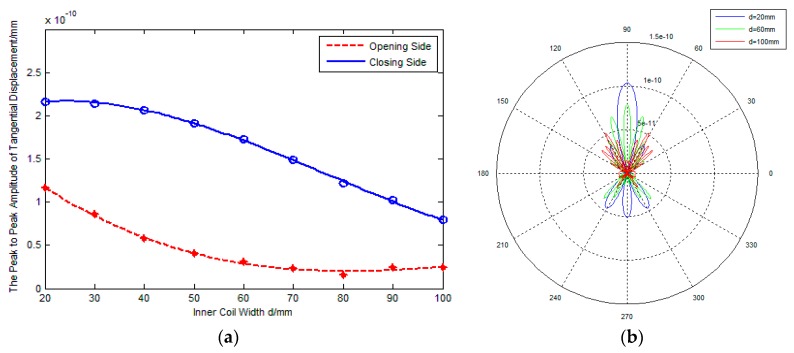
Influence of *d* on performance of PPM EMATs: (**a**) *d*-signal amplitude; (**b**) *d*-radiated acoustic field.

As shown in Figure 1b, the inner coil width *d* is defined as the distance between the inner line sources along x-axis due to line sources rotating an angle. Figure 3a,b show the influence of the inner coil width on the amplitude and directivity of excitation signals respectively, where the spacing between adjacent line sources ξ=2 mm and the rotation angle of line sources $\beta_{m}=45{}^{\circ}$.

In Figure 3a, the blue solid line represents the amplitude of excitation signals on the closing side of line sources, the red dashed line represents the amplitude of excitation signals on the opening side of line sources. It is found that the amplitude of the signal on the closing side is bigger than that of on the opening side. This illustrates that, compared with traditional PPM EMATs which excite waves equally in both sides, rotating parallel line sources can excite unidirectional waves to a degree. Furthermore, Figure 3a shows that the variation of the inner coil width affects the transducing efficiency greatly. For example, there is 22.7% decrease in the peak-to-peak amplitude of the signal on the closing side when the inner coil width increases from 20 mm to 60 mm, and the decrease of the signal amplitude on the opening side is about 73.9%. The main reason is probably that increasing the inner coil width can weaken the amplitude superposition effect and increase the diffusion attenuation, which decreases the amplitude of excitation signal. Namely, increasing the inner coil width can decrease the effective detection distance of PPM EMATs.

Figure 3b shows the radiated acoustic field directivity with variation of the inner coil width. It is found that the amplitude ratio $R_{ms}$ between the main lobe and side lobe decreases monotonously. This illustrates that the directivity of excitation signal is weakened with increasing of the inner coil width. It is probable that the waveform diffusion of excitation signal becomes more severe due to the decrease of the superposition effect while the inner coil width increases.

### 3.3. Effect of Adjacent Line Sources Spacing on Performance of PPM EMATs

**Figure 4:**
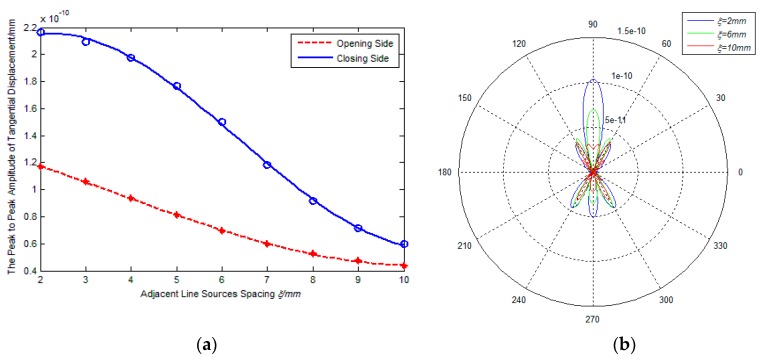
Influence of ξ on performance of PPM EMATs: (**a**) ξ-signal amplitude; (**b**) ξ-radiated acoustic field.

Figure 4a,b show the effect of adjacent line sources spacing ξ on the amplitude and directivity of excitation signal, where the inner coil width d=20  mm and the rotation angle of line sources $\beta_{m}=45{}^{\circ}$. As shown in Figure 1b, the overall width D of coil can be calculated by the formula $D=d+2\left(N-1\right)\left(\xi /cos\beta_{m}\right)$, where N is the turns of the coil, N=5. d and $\beta_{m}$ are the constant in this section. From Figure 4a, it is found that the amplitude of excitation signal decreases noticeably with an increasing in adjacent line sources spacing. For example, if ξ changes from 2 mm to 6 mm, there is 31.8% decrease in the peak-to-peak amplitude of the excitation signals on the closing side, and there is 42.5% decrease in the amplitude of excitation signals on the opening side. The main reason is probably that the increase of adjacent line sources spacing can lead to lower eddy current and therefore smaller Lorentz force amplitudes. It is indicated that increasing the spacing of adjacent line sources can decrease the effective detection distance of PPM EMATs. The phenomenon that the ratio $R_{co}$ decreases monotonously with the increase of adjacent line sources spacing indicates that increasing the adjacent line sources spacing can weaken the unidirectional excitation of transducers.

Figure 4b shows the radiated acoustic field directivity with various spacings of the adjacent line sources. It can be seen that the ratio $R_{ms}$ between main lobe and side lobe decreases monotonously with an increasing adjacent line sources spacing. The reason is probably that the waveform diffusion of excitation signals becomes more severe due to the decrease of the superposition effect. Namely, the directivity of excitation signal is weakened with the increase of the adjacent line sources spacing.

### 3.4. Effect of Rotation Angle of Line Sources on Performance of PPM EMATs

**Figure 5:**
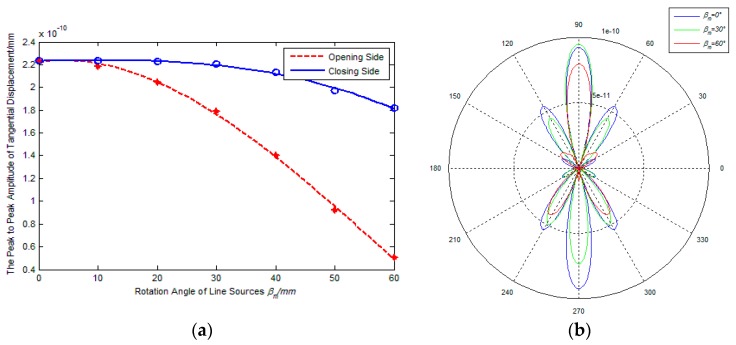
Effects of $\beta_{m}$ on performance of PPM EMATs (*d* = 20 mm); (**a**) $\beta_{m}$-signal amplitude; (**b**) $\beta_{m}$-radiated acoustic field.

**Figure 6:**
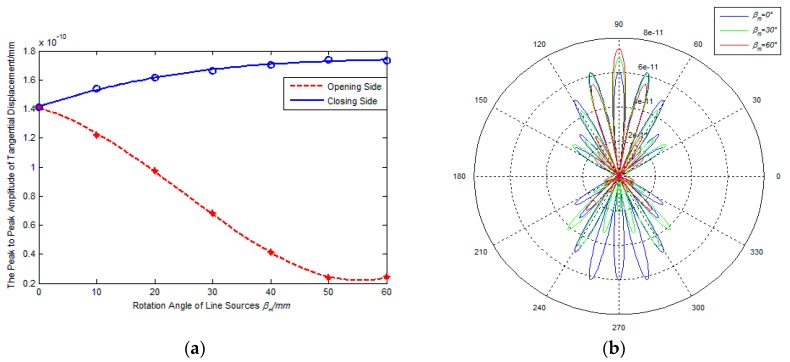
Effects of $\beta_{m}$ on performance of PPM EMATs (*d* = 60 mm): (**a**) $\beta_{m}$-signal amplitude; (**b**) $\beta_{m}$-radiated acoustic field.

Figure 5 and Figure 6 show the effect of rotation angle of line sources on the amplitude and directivity of excitation signal, where the adjacent line sources spacing is 2 mm and the inner coil width is 20 mm and 60 mm, respectively. From Figure 5a and Figure 6a, it is found that the amplitude of excitation signals on the opening side decreases with a continuously increasing $\beta_{m}$. However, the signal amplitude on the closing side shows different changing with the increasing of the $\beta_{m}$, and the amplitude decreases slightly if the ratio k=d/λ=1.25, the amplitude increases gradually if the ratio k=d/λ=3.75. The reason is probably that the inner coil width is the main impact factor for amplitude superposition effect with a relatively small value of k inner coil width and the wavelength, and the rotation angle of line sources becomes the major factor with the increase of the value of k. In addition, if the rotation angle of line sources changes from 0° to 60°, the signal amplitude gap between the closing side and the opening side increases continuously. It is indicated that the capacity of unidirectional excitation can be enhanced if increasing the rotation angle of line sources.

Figure 5 and Figure 6b show the radiated acoustic field directivity if the rotation angle of line sources is 0°, 30°and 60° respectively. From Figure 5b, it is found that the amplitude of main lobe on the closing side is smallest if the rotation angle of line sources $\beta_{m}$ = 60°, but the amplitude ratio $R_{ms}$ between main lobe and side lobe and the amplitude ratio $R_{co}$ between closing side and opening are largest. This illustrates that increasing the rotation angle of line sources can’t enhance the amplitude of excitation signal significantly if the inner coil width is relatively small, but it can improve the unidirectional and directivity of transducers. From Figure 6, it can be seen that the amplitude of main lobe on the closing side increases gradually with an increasing in the rotation angle of line sources, and the variation tendency of main lobe on the closing side is in contrast to that on the other side. This illustrates that the rotation angle of line sources is the main impact factor for the amplitude superposition effect while the inner coil width being relatively large. That is to say, increasing the rotation angle of line sources can enhance the amplitude of excitation signals, the capacity of unidirectional excitation and directivity of transducers. During comparison with Figure 5b, Figure 6b shows that the amplitude ratio $R_{ms}$ between main lobe and side lobe decreases and the number of side lobes increases if the inner coil width increases at the same rotation angle. Apparently, the directivity of excitation signal can be weakened with an increasing inner coil width, which is same as the conclusion drew in Section 3.2.

## 4. Experimental Analysis

**Figure 7:**
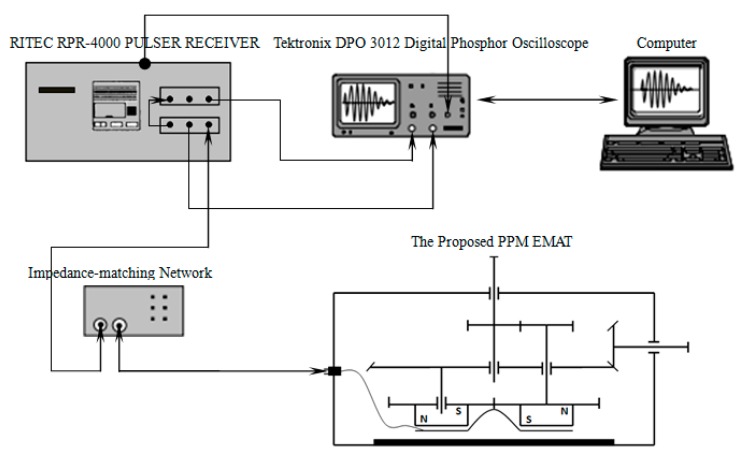
The experimental setups.

According to the above analysis, a PPM EMATs, shown in Figure 7, which can focus and revolve the SH wave is developed based on the simulation results. The experimental setup includes a RITEC RPR-4000 Pulser Receiver, a Tektronix DPO 3012 Digital Phosphor Oscilloscope, a 3 mm aluminum plate and the PPM EMAT. The impedance matching is realized by paralleling a capacitance with the coil. In PPM EMATs, rotating the wave-beam-focused mechanism will change the angle between parallel line sources. Turning the wave-beam-revolved mechanism can make PPM and racetrack coil rotate, which changes propagation direction of the SH wave.

Three coils, which parameters shown in Table 2, are excited by the RITEC RPR-4000 in terms of the pulse echo reflecting method respectively. The geometrical structure of Coil I and Coil II is that of a conventional PPM EMAT, Coil III is the proposed PPM EMATs which line source rotation angle is 30°.

**Figure 8:**
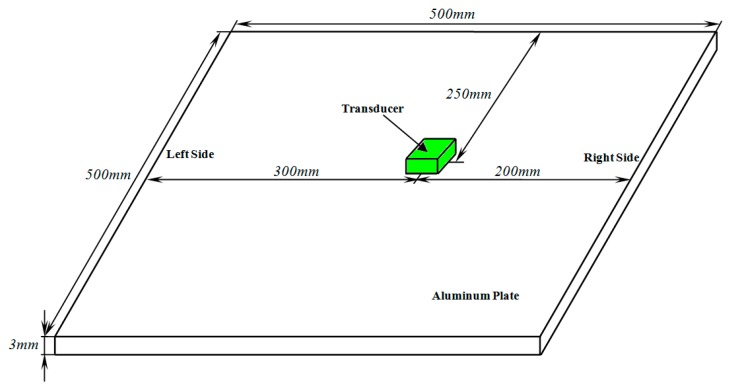
The experimental configuration.

Figure 4a shows that the amplitude ratio $R_{co}$ between closing side and opening side reaches the best level when the inner coil width ranges from 50 mm to 70 mm, so the inner coil width of coil III is defined as 70 mm in the experiment. As shown in Figure 8, the distance between each coil and the right end of aluminum plate are the same. Additionally, in order to avoid the superposition of the reflected echo from the two ends of the aluminum plate, the distance of each coil to the right end of aluminum plate is shorter than that to the left end. Owning to the bilateral symmetry of Coil I and Coil II, the echo on the right side of aluminum plate can be considered equal. Rotating Coil III 180°, the echo on the right side of aluminum plate generated by both sides of Coil III can be measured, respectively.

**Figure 9:**
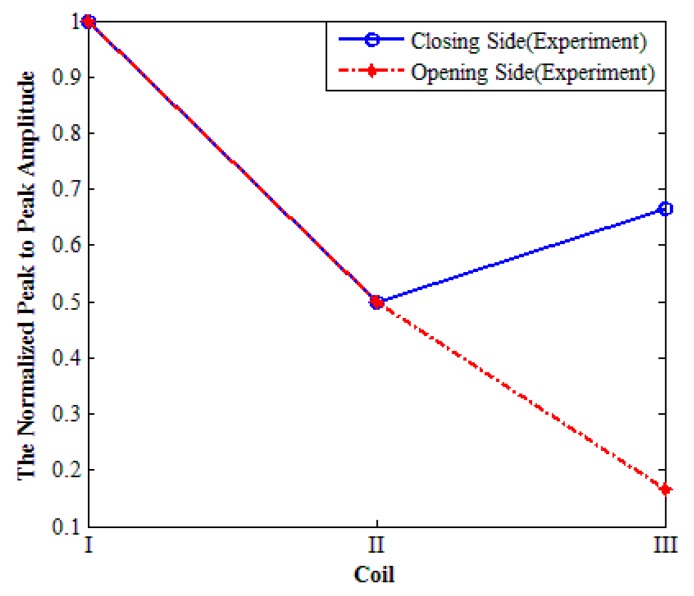
The normalized peak-to-peak amplitude of echo on the right end of aluminum plate.

The peak-to-peak voltage of echo, detected by the oscilloscope, from the right side of aluminum plate is measured in the experiment, which is shown in Figure 9. The experimental data in Figure 9 are normalized by the peak-to-peak amplitude of Coil I respectively. From Figure 9, it can be found that the signal amplitude of Coil II is lower than that of Coil I, which indicates that increasing the inner coil width can weaken the effective detecting distance of the transducer.

Compared with Coil II, the signal amplitude of Coil III is higher. This illustrates that rotating the parallel line sources can suppress the negative influence caused by inner coil width increasing. Apparently, the $R_{co}$ of Coil III is much higher than that of Coil I and Coil II, so it can be concluded that the unidirectional excitation ability of Coil III is much higher than that of Coil I and Coil II. Namely, rotating the parallel line sources can enhance the unidirectional excitation ability of PPM EMATs. The phenomenon that the signal amplitude of Coil III is lower than that of Coil I may results for the following reasons: in the experiments it should be noticed that the orientation of line sources of Coil I and Coil II is vertical to the vibration direction of particles, but there is an acute angle between the orientation of line sources of Coil III and the vibration direction of particles. The acute angle may weaken the amplitude of echo detected by Coil III. Additionally, the electrical impedance matching can be adjusted discretely by replacing the capacitance paralleled to the coil. It is very difficult to make sure that the electrical impedance matching reaches the best condition accurately. Thus, the electrical impedance matching may also enlarge the disagreement.

## 5. Conclusions

A PPM EMAT which can focus and revolve the SH wave is proposed in this paper. The effects of parameters such as inner coil width, adjacent line sources spacing and rotation angle between parallel line sources on SH wave focusing and directivity are investigated. The simulation and experimental results demonstrate that rotating the parallel line sources can strength the wave beam on the closing side of line sources and reduce that on the opening side. Moreover, both decreasing the inner coil width and reducing the adjacent line sources spacing can enhance the amplitude and directivity of excitation signal. Compared with traditional PPM EMATs which excite waves equally on both sides, the capacity of unidirectional excitation and directivity of PPM EMATs proposed in this paper has been improved significantly.

## Figures and Tables

**Table 1:** The size and material parameters of the aluminum plate.

Parameter	Value	Parameter	Value
Length	500 mm	Conductivity	3.5E-7 S/m
Width	500 mm	Elasticity modulus	70 GPa
Thickness	3 mm	Poisson’s ratio	0.33
Density	$2700\;\mathrm{kg}/m^{3}$		

**Table 2:** The geometrical parameters of the coils.

	Inner Coil Width (*d*)	Rotation Angle of Line Sources ($\beta_{m}$)	Spacing of Adjacent Line Sources (ξ)	Turns of Coils (*N*)
Coil I	10 mm	0°	0.905 mm	25
Coil II	70 mm	0°	0.905 mm	25
Coil III	70 mm	30°	0.905 mm	25
